# Supplementary material for: Understanding ambivalence in help-seeking for suicidal people with comorbid depression and alcohol misuse
Source: PLoS One. 2020 Apr 17;15(4):e0231647. doi: 10.1371/journal.pone.0231647 (PMC7164619; doi:10.1371/journal.pone.0231647)
Supplement: Attachment — Submitted filename: 130619 - response to reviewers.docx [file pone.0231647.s002.docx]

**REVIEWER 1 – please see Reviewer Comments in bold and our response in** normal text.

**The authors present a well-written qualitative study of suicidal behavior among people experiencing depression and alcohol/substance use. The analytic insights contain a few quite promising leads, such as the “paradox of engagement,” wherein participants wanted to be self-reliant yet expected others/health systems to reach out and help them. The authors’ identification of complexities with regard to social support systems and suggested use of alternative forms of support for individuals avoiding formal ones are well-received. I do think, however, that the paper needs a bit more revision before being suitable for publication.**

We thank the Reviewer for this thoughtful reflection on the manuscript and for the comments that follow. We trust we have addressed them adequately.

**First, there is an issue with the nature and organization of findings. With qualitative research of this kind that, as you mentioned, attempts to describe participants’ experienced meanings, the overarching themes should reflect such experienced meanings as directly as possible. As it stands now, however, some of the themes do not reflect their experienced meanings, but more seem to reflect the researcher’s frame and understandable intention to enhance help-seeking behaviors. For instance, “mode of help-seeking”, “barriers to help-seeking”, and “comorbidities” are themes that do not appear to reflect participants’ directly experienced meanings. From my reading, it appears that almost no one in this sample reached out for support, which would suggest that there was little to no help-seeking involved (at least based on the data presented). The realm of others were experienced more along the lines of shaming and dismissive, with the participant feeling like a burden to them. These experiential parts were placed under your heading of “barriers to help-seeking”, but it does not seem that, for many, they tried to reach out and then experienced barriers (some did experience it this way, but not all). For most, it seemed that there was a basic deprivation of support, exacerbated by logistical, financial, and geographical structures, all of which presented a context in which participants rarely considered help. Even the notion of “help” needs to be explored more, perhaps with reference to the circumstances that led them to consider suicide in the first place. I imagine that participants wanted “help” for many things, including with aspects of their life circumstances (e.g., more help with childcare).**

This is an important insight, and we thank the Reviewer for these reflections. We have gone back to the data provided by participants and sought to more accurately apply thematic meaning to their experiences as per their utterances, rather than from the higher order labels that we (as healthcare providers and researchers) might use to categorise these experiences. For example, we have reframed “mode of help-seeking” to “affordability, access, and awareness of services”, “barriers to help-seeking” to “reluctance to seek help”, and “comorbidities” to “compounding life events and coping mechanisms”. We have retained the theme of “paradox of engagement in help seeking” and attempted to expand on this paradox. We have attempted to represent, from the participants’ experience, what these themes represent, rather than interpret (using our own frameworks) what we thought they were referring to.

**One main purpose of making these qualitative distinctions and of remaining close to the original experience is to go beyond what is already conceptually known about the topic, in order to uncover insights that may be currently obscured by our own frames of theorizing. After all, it is clear that the mental health system was not a suitable option for these participants, so research that remains closer to their actual experience is better positioned to close the gap between everyday community life and mental health systems. The section on paradox of engagement gets much, much closer to the way they experience it and was a quite good section overall (the summary at the lead of the Discussion was also quite good and near-to-experience in this respect). In all, though, there needs to be much more attempt at reflecting the structure of the experience as lived, replete with descriptions of essential meanings.**

Again, we thank the reviewer for these valuable insights, and have tried to reframe the data using the participants’ own utterances rather than our interpretation of what this relates to in the broader healthcare literature and context. We have applied Bronfenbrenner’s ecological systems theory to the interpretation of the main findings of the study, and have attempted to ensure that this interpretative discussion is separated out from the results of the study.

**Second, the concrete rationale for this specific study needs to be better developed. As of now, it appears as a more global connection between previous literature and this study—which is a bit too general for a journal of this type. One way to correct this is, I believe, if the authors conduct a more concrete critique of the previous literature, which would then set up the specific need for this current study. There are some gaps otherwise: For instance, why focus on suicide attempts? Why focus on help received and desired? It may seem obvious but filling in these gaps would greatly aid both the organization and readability of your paper.** **On a related note, I find the following statement hard to believe: “Despite this, little research exists on the relationship between alcohol use and suicidal behaviours.” Most practicing clinicians are well-aware of this link, as it is also reflected in most suicide prevention guidelines. Presumably these guidelines come not only from years of clinical experience but also from research (including case studies). I imagine many readers will also wonder about this, so I would suggest that the authors at least mention some of the available research or guidelines, and then suggest a reason as to why more research is needed.**

We have added literature and a consideration of the nature of suicidality into the introduction, and removed the statement about alcohol use and suicidal behaviours. To clarify, however, whilst clear data have demonstrated the links between suicidal behaviours and alcohol misuse, much less has examined help-seeking for suicidal ideation/attempts in people with alcohol misuse problems. We have also presented a discussion of why more research is needed in this area.

**Once the above two issues are addressed, I believe that the authors will be in a place to offer even more specified and novel suggestions for policy, practice, and action. Some of the ones mentioned are of course good and thoughtful, such as the focus on alternative means of support and ways to address the “burden” issue. But I believe more can be said about ways to combat stigma, isolation, and hopelessness, such as access to stories of recovery or the involvement of peers who have been through it. And again, we need to know more about the life circumstances that are leading them to consider suicide in the first place, which may ultimately also implicate the depriving social world around them.**

Our interviews did not explore the life circumstances leading participants to consider suicide in the first place, which is a limitation of the current study (reflected in the Discussion). We have applied Ecosystems theory to the study results, which attempts to understand (and place) the role of peers, family, and other support systems in the life of our participants leading up to their suicide attempt. We believe that applying this lens deepens the insights gained from this piece of work.

**I would explain more about your view of what “data saturation” entails. I generally remain a bit uncomfortable with this idea of saturation, given that more can always be said or explored about a given topic, even within one participant. Further, your sample appears to be quite acculturated to mainstream culture. So, I would personally avoid suggesting that saturation means that your findings are all that can be said about the topic.**

We have removed the term ‘data saturation’ from the manuscript, as we agree with the Reviewer’s comments in relation to the current sample. We have addressed the sample size as a limitation in the revised manuscript.

**On a related note, there’s a need to speak on limitations with regard to excluding people with an ‘inability to comprehend English’. I’d imagine these folks are doubly suffering from social isolation and are in great need of support.**

This has indeed been added to the limitations section in the discussion.

**Kindly clarify if the 4 standard drinks is the recommended limit or exceeds the limit—the writing is a bit unclear.**

Four standard drinks is the recommended limit and drining in excess of this limit was the basis for inclusion in the study. This has been clarified.

**There are a handful of typos which can be found upon another read-through.**

We hope we have addressed these

**I was unsure what the restrictions on data access were, or if these need to be stated.**

Thank you for this point. Given the limits of our ethics approval for the study, and the identifiable nature of the interview transcripts, we are unable to provide open access to the data for the current study. Instead, we can provide access to the transcripts by application via our Human Research Ethics committee, and have provided details about this accordingly.

**Overall, this paper holds promise and is in a much-needed area of study. My suggestion is for the authors to return to their participants’ experience again, to reflect its internal structures more closely, all in the attempt to close the gap between those in need and the supports that could perhaps help them.**

Thank you for this comment. We hope that in doing this, we have been able to address (and adequately reflect) the Reviewer’s comments on the manuscript.

**REVIEWER 2 – please see Reviewer Comments in bold and our response in** normal text.

**The manuscript "A qualitative analysis of suicide attempts made by people with comorbid depression and alcohol use disorders" fills a necessary gap in the literature. It can potentially provide a way to understand the intersection of these three phenomena from users' point of view and assist in guiding treatment. The manuscript is well organized and the writing is clear. However, in its current form, the manuscript requires a significant revision to improve the method and make the results clearer to the reader. In general, the methods section suffers from an overly cursory approach to qualitative methods, and often does not seem theoretically coherent, which makes assessing the rigor and the manuscript difficult.**

Thank you for this comment. We have engaged additional qualitative experts to assist in the interpretation of the study data, and hope that this revision addresses these issues.

**Methods section: The rationale for thematic analysis is not clear to the reader. There are a variety of methods that target the meaning of participant's experience, some more explicitly than thematic analysis. As the interview questions aimed for participants explanations of behavior, it is unclear how the researchers came to understand the meaning of participants experience. (This issue is reflected in the results/themes as well.) The discussion of stories versus formal models is unclear, especially with respect to how a narrative analytic approach was integrated with thematic analysis. To be clear, it is not that the authors should choose one approach, rather the method could be better explicated in order for the reader to be able to clearly understand the rationale and analytic process. The final sentence of the method section also is unclear yet important and deserves more attention. How did the authors understand "richness?" This could be unpacked further and is dependent on greater clarity at the outset of this section. Specifically, being clear about one's rationale will guide how richness is understand. There also is a variety of literature that the authors can draw from to further explain this area (e.g., catalytic validity), but, the literature depends on the analytic approach chosen.**

Thank you for these comments. They have made us think deeply and analytically about these interviews and the analysis we have undertaken. We have made significant amendments to the study methods and results sections of the manuscript, and we believe this addresses the Reviewer’s concerns about clarity, richness, and interpretation of the results.

**Results: The results section reads more like a summary of participants' responses rather than an integrated and coherent set of themes. For instance, the authors seem to undue each theme at the end of many of the sections. Divergent experiences (e.g., finding community-based care satisfying) should be integrated into the theme or removed. This issue may be resolved through a reconceptualization of the superordinate theme. Additionally, as written, the results do not convey the meaning, or how people experience a phenomena. For instance, it is clear that participants had difficulty disclosing suicidal thoughts/hopelessness, but it is unclear what meaning this holds for people.**

Thank you for these comments, and we agree. As per the response to Reviewer 1 above, we have significantly re-conceptualised the themes arising from this set of interviews, into (what we belive) is a much clearer set of themes, driven by the participants’ words and experiences directly, rather than our interpretation of what we believe they were communicating.

**A few thoughts:

Social support: 1. In the final paragraph of page 12, the sentence "This then created stigma…" is unclear. How was stigma created? 2. Something feels missing here. How do the participants move from not wanting to disclose to people they know to being willing to in the future?**

**In both social support and community-based services there was a reluctance to disclose yet it manifested differently. The authors may want to frame this section around this finding. "Mode of help seeking" doesn't seem to fit.**

**It is not always clear to the reader how the description and the quotations relate.**

**Comorbidities may not be the best title for this section. It may inadvertently pathologize life experiences (like breakups, loss), as comorbidities connotes concurrent health conditions.**

We agree with the above thoughts of the Reviewer, and believe that our re-conceptualisation of the study results has addressed these concerns. As per our comments to Reviewer 1, we have reframed “mode of help-seeking” to “affordability, access, and awareness of services”, “barriers to help-seeking” to “reluctance to seek help”, and “comorbidities” to “compounding life events and coping mechanisms”. We have retained the theme of “paradox of engagement in help seeking” and attempted to expand on this paradox. We believe we have more clearly linked and integrated themes, descriptions of themes, and participant quotations that exemplify each theme. We have left analysis and interpretation of these themes in a broader sense to the discussion section.

**Limitations. The treatment of bias here can be elaborated by addressing it from within the qualitative tradition rather than experimental approaches. It may be useful to address bias by discussing research reflexivity and how, rather than if, the results were biased. Earlier in the document, how it was decided that saturation was reached could be better explained.**

These are excellent points. We believe that our revision of the limitations section (and other sections) of the manuscript addresses these issues.

**Future directions. Do not seem to follow from the results.**
This section has been removed, and replaced with a “Conclusions” section which we believe follows directly on from the results of this study.
