# Supplementary material for: Understanding ambivalence in help-seeking for suicidal people with comorbid depression and alcohol misuse
Source: PLoS One. 2020 Apr 17;15(4):e0231647. doi: 10.1371/journal.pone.0231647 (PMC7164619; doi:10.1371/journal.pone.0231647)
Supplement: Attachment — Submitted filename: Response to Reviewers.docx [file pone.0231647.s003.docx]

**Response to Reviewers:**

Thank you for taking the time to review our manuscript and provide such thoughtful feedback on our work, which has helped to strengthen our paper. Please see our response to each point raised by the reviewer below. We welcome any further feedback on our revisions.

**Reviewer #2:** In my opinion, the main flaw of this paper is the attempt of the authors to make general inferences from very limited qualitative data to support their theory in the Discussion and Conclusion sections. For example,

At the end of the Conclusion you state that “Application of an ecological systems perspective confirmed the significance of support at the microsystemic level. It also illuminated the critical link between attitudes and behaviours at the micro level, and constructions of suicide at the broader macro level. Recognising and addressing the cascading influence of broader socio-cultural perspectives on suicide, mental health and comorbidity, is crucial to ensuring the effectiveness of future intervention and prevention measures.” I do not think your qualitative data “confirms” these conclusions and I did not see any evidence from the data to support a “critical link” between the micro and macro level. Perhaps you could say something like: “the qualitative data revealed themes that illustrated many challenges of engaging with different support systems which lends itself well to an ecological systems theory perspective when considering interventions and approaches for this complex population.”

**Author response:** Thank you. We agree with your feedback and have amended our wording in line with your suggestion.

**Reviewer #2:** Similarly, in the last paragraph of the Discussion Section, you make statements that are too broad and do not justify the data, e.g.: “The key finding of this study, that individuals experiencing acute phases of suicidality do not typically access traditional treatment services and are reluctant to seek support from family and friends, is consistent with findings from a number of previous studies (Fogarty et al. 2018). While this finding highlights the importance of support and engagement at the micro and meso-systemic levels, it also illuminates the critical role of broader social and cultural macrosystemic – factors in shaping people’s attitudes and behaviours” I do not think that is the “key finding” of this study based on the qualitative data you presented. In my opinion, your study illustrates the ambivalence and different types of challenges people experience seeking help when they are suicidal when they engage with different systems of support. In addition, you state in an earlier paragraph in the Discussion Section that people are more likely to seek help from family and friends rather than professionals which contradicts this last paragraph where you introduce the “key finding” that e people are reluctant to seek help from family and friends.

**Author response:** Thank you for making us aware of these issues. We have revised this paragraph to address the inaccuracies and the contradicting statements you refer to (see p. 25).

**Reviewer #2:** I don’t find the concept “paradox of service engagement” very helpful and I don’t think it does justice to your data. My impression from your data is that many of these individuals had significant ambivalence asking for help due several factors such as negative experiences with family or formal providers or fears about how these different groups would respond if they reached out for help and disclosed being suicidal. I would prefer more descriptive words in your title and abstract that are closer to the data such as the words “ambivalence” or “challenges” rather than the impression that you have discovered a whole new concept called “paradox of service engagement” – again similar to the Discussion and Conclusion sections it seems like you are trying to reify concepts from qualitative data which does not lend itself to such generalizations.

**Author response:** Upon reflection, we agree that the “paradox of engagement” does not do justice to our data. We have removed this expression from the manuscript (including from the title and abstract) and have instead used descriptive words that are closer to the data (see p. 1, 2, 19, 23, 26).
